# Alzheimer’s disease as a women’s health challenge: a call for action on integrative precision medicine approaches

**DOI:** 10.1038/s44294-024-00021-3

**Published:** 2024-05-20

**Authors:** S. Miramontes, C. Pereda Serras, S. R. Woldemariam, U. Khan, Y. Li, A. S. Tang, E. Tsoy, T. T. Oskotsky, M. Sirota

**Affiliations:** 1 Bakar Computational Health Sciences Institute, University of California San Francisco, San Francisco, CA USA; 2 Memory and Aging Center, Department of Neurology, University of California San Francisco, San Francisco, CA USA; 3 Global Brain Health Institute, University of California San Francisco, San Francisco, CA USA; 4 Department of Pediatrics, University of California San Francisco, San Francisco, CA USA

**Keywords:** Diseases, Diagnosis, Therapeutics

## Abstract

Alzheimer’s Disease (AD) is marked by pronounced sex differences in pathophysiology and progression. However, the field has yet to fully recognize AD as a women’s health issue, delaying the development of targeted preventative strategies and treatments. This perspective explores the elements impacting AD in women, identifying sex specificity in risk factors, highlighting new diagnostic approaches with electronic health records, and reviewing key molecular studies to underscore the need for integrative precision medicine approaches. Established AD risk factors such as advancing age, the apolipoprotein E4 allele, and poorer cardiovascular health affect women differently. We also shed light on sociocultural risk factors, focusing on the gender disparities that may play a role in AD development. From a biological perspective, sex differences in AD are apparent in biomarkers and transcriptomics, further emphasizing the need for targeted diagnostics and treatments. The convergence of novel multiomics data and cutting-edge computational tools provides a unique opportunity to study the molecular underpinnings behind sex dimorphism in AD. Thus, precision medicine emerges as a promising framework for understanding AD pathogenesis through the integration of genetics, sex, environment, and lifestyle. By characterizing AD as a women’s health challenge, we can catalyze a transformative shift in AD research and care, marked by improved diagnostic accuracy, targeted interventions, and ultimately, enhanced clinical outcomes.

## Introduction

Numerous studies have highlighted sex differences in Alzheimer’s Disease (AD), emphasizing that women bear a significantly higher risk compared to men. By age 45, women face a 1 in 5 lifetime risk of AD, whereas men have a 1 in 10 risk^1^. Currently, almost two-thirds of individuals living with AD in the US are women^1^. Despite widespread acknowledgment of these statistics, the field has yet to recognize AD as a women’s health issue and adopt a proactive stance in developing targeted treatments, formulating preventative strategies, and refining the clinical identification of at-risk women to improve AD diagnosis. In fact, this gap in proactive measures echoes historical patterns. For example, heart disease stands as the leading cause of death^2^, but “canonical” symptoms such as chest pain in acute myocardial infarction apply disproportionately to men, with women more likely to have “noncanonical” symptoms like fatigue and weakness^3^. We now know that there are sex differences in not only symptoms, but also risk factors, treatment, and mortality, highlighting the importance of considering sex in disease. To illustrate, a recent phase 3 clinical trial of lecanemab, a monoclonal antibody therapeutic for AD, demonstrated slowing of cognitive and functional decline for participants of this study who were treated with this drug^4^; however, this effect was more pronounced in male and not female study participants^4^. In this perspective, we spotlight the clinical and non-clinical factors that impact the prevalence of AD in women, explore contemporary diagnostic approaches using electronic health records (EHR), and delve into sex differences in AD revealed by molecular studies. This underscores a critical call to action in advancing the field of precision medicine in AD. We advocate for research aligned with the biological recognition of female sex within the pathophysiology of AD, pushing towards patient-centered prevention, diagnosis, and treatment strategies.

## Definition of sex and gender

We define sex as the biological difference between females and males with chromosomes XX and XY, respectively, and gender as a person’s psychosocial and cultural self-identification as being a woman, a man, or other identities. Note that one’s gender may exist on a spectrum and not align with biological sex. We recognize the critical importance of considering health outcomes in transgender, non-binary, and intersex communities. Thus, we include information addressing the differences in AD among individuals identifying as women (trans-women) with and without the chromosomal complement XX. Hereafter, we refer to cisgender women and cisgender men as “women” and “men”, respectively.

## AD biological, clinical, and sociocultural risk factors

Although the underlying causes of AD remain unclear, it is now widely understood that early symptom recognition and risk factor management can potentially improve clinical outcomes^5^. Primary risk factors for AD include advancing age, carriage of the apolipoprotein E-ε4 (APOE-ε4) allele, and female sex^1^—females aged 80–99 years show significantly higher prevalence rates for AD compared to males of the same age group^6^. Female hormonal changes, particularly during menopause, have also been linked to an elevated risk of dementia^7,8^. Among modifiable factors, cardiovascular risk factors like hypertension, high cholesterol, and diabetes have a more pronounced negative impact on AD in women compared to men^9^. Depression, an AD risk factor for both cisgender men and women, disproportionately affects women, with a twofold likelihood of incidence compared to men^10^. Emerging research also highlights that non-binary adults (49.8%) and transgender men (30.7%) are disproportionately affected by depression when compared to cisgender men (13.8%) and cisgender women (23.6%)^11^. Sociocultural risk factors, such as unequal access to formal education, employment and occupational opportunities^12^, may contribute to gender differences, as they have persisted through this century. A study in the UK found that low formal educational attainment was associated with an increased risk of dementia mortality in women but not in men^13^. Occupational inequities have also been reported: A recent study found that women who engaged in paid work earlier in life exhibited better cognitive outcomes after age 60 compared to those who did not participate in the paid workforce^14^. Additional gender disparities emerged during the initial stages of the COVID-19 pandemic, including heightened responsibilities in childcare and increased job losses in sectors predominantly occupied by women^15^. While the impact of these disparities on brain health remains unclear, researchers are investigating how challenges in mental health, job opportunities, and reduced earnings during the pandemic may influence women’s brain health throughout adulthood^16^. These and other gender inequalities remain critically understudied in low- and middle-income countries (LMIC), where they are likely to be even more consequential given the rapid global increase in life expectancy among women compared to men^17^. By proactively identifying sociocultural and clinical risk factors, we can empower healthcare professionals and policymakers to deploy targeted interventions promptly. This approach is geared towards risk reduction, leading to an enhanced quality of life for individuals developing AD^1^.

AD diagnosis and care in women therefore requires careful consideration of sex and gender differences. Medical history can provide valuable insights into clinical risk factors and/or potential genetic predisposition. To illustrate the clinical sex differences between males and females, a recent study performed deep phenotyping of AD-generated comorbidity networks between patients with AD in a sex-stratified manner. The findings provided a phenotypic representation of disease interactions among patient groups, highlighting significant differences between males and females, with females showing a stronger effect size with depression, hypertension, urinary tract infections, and osteoporosis^18^—all aligning with prior findings. Further applications of machine learning to identify patients at higher risk of AD have shown better predictive power in female patients’ data relative to males, and have found known and novel sex-specific associations^19^.

Findings from biomarker testing may also differ between male and female AD patients, and understanding these nuances is essential for accurate diagnosis and personalized treatment plans. Biomarker tests are crucial for early detection and monitoring disease progression, and sex differences in biomarker profiles must be considered, as these tests are increasingly utilized in healthcare settings^20^. Several studies report higher cerebrospinal fluid (CSF) and plasma phosphorylated tau levels in females on the AD continuum in comparison to males^21^. Although no sex differences have been shown in amyloid-β (Aβ) concentration in CSF nor Aβ positron emission tomography positivity in AD patients^22^, clinically normal females with higher Aβ burden have been shown to have higher tau levels^23^ in the entorhinal cortex, one of the most vulnerable brain regions in AD. Furthermore, Aβ-positive female patients exhibited a faster cognitive decline^21^ in association with higher baseline plasma phosphorylated tau levels compared to males. As research is ongoing to identify and evaluate reliable biomarkers for AD, the increasing evidence of sex effects on AD biomarkers supports the need for considering sex in the diagnostic process.

## The effect of APOE4 on AD risk and biomarkers

Sex differences in biomarkers are further influenced by APOE-ε4 genotype, the most established genetic risk factor for AD: prior studies show that the association between APOE-ε4 and CSF tau levels are stronger in females relative to males^24^. APOE genotype also affects AD risk and disease progression in males and females differently^25^. The risk of AD onset or conversion from mild cognitive impairment (MCI) to Alzheimer’s dementia has been reported to be higher in female APOE-ε4 carriers than in male carriers^22^. Female APOE-ε4 carriers with MCI have also demonstrated greater hippocampal atrophy than male carriers with MCI^22^. Furthermore, an analysis of over 1500 AD patients’ serum discovered female APOE-ε4-carrier-specific metabolites that did not appear in males or in females without APOE-ε4^26^. These studies suggest a specific effect of APOE-ε4 on the female sex, and further emphasize the importance of precision medicine approaches in the clinical implementation of biomarkers and genetics in AD.

## Biological hypotheses behind sex and gender differences—multiomics and beyond

Sex differences are a major source of disease heterogeneity in AD^27–32^. Although vulnerability to genetic load and severity of AD pathological burden have been well established, differentially mediated molecular underpinnings and pathways between male and female AD patients are still poorly understood. Using omics and integrative computational approaches to study the molecular mechanisms behind sex dimorphism in AD can aid in the advancement of targeted treatments^33^. A meta-analysis of over 1000 patients’ transcriptomes unveiled a female-specific differential expression of adaptive and innate immunity-related genes, as well as downregulation of signaling pathways related to synaptic vesicle exocytosis and autophagy^19^. Single-cell studies have enabled cell type investigation of sex-specific disease signatures: in the prefrontal cortex, glial cells exhibited differential expression unique to female AD brains in synaptic transport pathways, while in the entorhinal cortex, genes related to tau processing, protein folding, and phagocytosis were downregulated in microglia^34^. These sex-specific immune and inflammatory responses are likely contributors to disease heterogeneity in AD^35,36^. Multiomic studies, using transcriptomics or proteomics, can facilitate a better understanding of the differences in AD pathogenesis and clinical manifestations to help identify and stratify patients at risk^37^ for clinical trials and precise treatments.

Biological hypotheses to explain the molecular sex differences have centered around hormonal differences and X-chromosomal biology. As sex hormone levels decline with age, decreased exposure to estrogen has been associated with higher AD risk in female patients^38^. This could be in part due to a reported decline in hormonal receptors with age and higher expression of nonfunctional splicing variants of hippocampal estrogen receptors in females compared to males^33^. Bulk RNA sequencing studies have also shown that androgen and estrogen receptors disrupt the homeostatic processes of AD neurons^39^. Based on this hypothesis, hormone replacement therapy around menopause has been studied for its effect on cognitive decline in female AD patients, but large-scale trials have been mostly inconclusive^33^. In contrast, brain imaging studies have provided encouraging evidence for a positive association between greater cumulative estrogen exposure and lower AD risk in women^40^. Utilization of hormonal contraceptives (HC) was associated with a lower AD risk among women. Around 56% of studies investigating the impact of HC report a reduced risk of cognitive impairment^8,41,42^ or higher scores on cognitive tests in middle-aged women taking HC^8,43,44^. There are also reports of a 50% reduced risk of cognitive impairment in older women (60+ years) who had used HC compared to never users^8,45^. However, other studies reported no associations between HC use and dementia incidence, cognitive decline, or cognitive performance, which may be due to several factors including age of initiation, HC formulations, dosage, and duration of use^8^.

Gender-affirming hormone therapy (GHT) facilitates the development of secondary sex characteristics more consistent with an individual’s gender identity, partly by reducing the characteristics of their biological sex at birth. Given prior evidence indicating potential negative impacts of hormone deprivation on cognitive aging and the risk of AD^8,46–49^, there is a question about whether GHT affects cognitive health in transgender individuals. Despite limited research in this area, meta-analyses suggest no adverse effects on cognitive function in young adult transgender men or women^8,50^. GHT effects on cognition in older individuals are less clear as only one study of transgender women has investigated the long-term effects of GHT, where transgender women performed similarly to cisgender men on all cognitive tests, but scored lower than cisgender women on memory tests (immediate and delayed recall)^8^.

Similarly, as men grow older, total testosterone concentrations generally decline. While several studies have reported on the associations between lower testosterone concentrations and poorer health outcomes in aging men, including cardiovascular events and mortality, evidence remains largely observational and mixed^51^.

On the other hand, as X-chromosomal inactivation increases in females with age, the complex role of the X chromosome has been increasingly studied for its potential contribution to AD in males and females. For instance, a bulk transcriptomic study of AD patients’ dorsolateral prefrontal cortex showed significant associations of X chromosome expression with tau pathology specifically in men^52^. Interestingly, female and male mice expressing human amyloid precursor protein with an additional X chromosome showed increased resilience to AD-related vulnerability^53^. Future studies can help clarify the role of the X chromosome in AD pathology in the context of biological sex.

## The impact of precision medicine on AD

Precision medicine is an emerging integrative approach for disease prevention, early detection, and treatment, which takes into account individual variability in genetics, epigenetics, sex, environment, and lifestyle. The current and ever-growing availability of public omics data of normal and AD brains, including Gene Expression Omnibus, Array Express, and the new National Institute on Aging Accelerating Medicines Partnership for AD portal (AMP-AD), along with computational tools to dissect molecular drivers of disease at a network level, present a unique new opportunity to explore sex differences in AD pathogenesis from a molecular multiomic perspective. Using proteomics^37^, metabolomics^26^, or transcriptomics^19,34^, new biological insights will enable greater precision in patient identification and stratification to improve treatment effectiveness. In addition, real-world datasets such as EHR provide an abundance of longitudinal clinical data, yet these datasets have yet to be optimally utilized to investigate sex-specific AD complexity. A recent study leverages EHR data and genomics to identify clinical predictors of AD and shared genetic associations underlying those predictors in a sex-specific manner^54^, elucidating how large datasets can be used to inform precision medicine approaches.

In the past two decades, these transformative breakthroughs and cutting-edge computational approaches have converged, offering an unprecedented opportunity to delve into and enrich our comprehension of sex differences in AD pathophysiology. This convergence not only paves the way for groundbreaking diagnostic and therapeutic strategies but also underscores the pivotal need to assess AD risk in women through both gender and biological lenses. Integrating multiomics, digital, and computational advances into precision medicine approaches will enable a more exhaustive and impactful pursuit of optimal treatments and facilitate improved outcomes in AD care. Other important directions include the development of precision medicine infrastructure in low-resource settings and the evaluation of its social and economic impacts around the world. These advances will require a collaborative effort among patients, clinicians, researchers, policymakers, healthcare systems, and the pharmaceutical industry who by working together to recognize and tackle these critical problems can advance precision medicine for all.
